# Development and validation of a questionnaire to evaluate the factors influencing training transfer among nursing professionals

**DOI:** 10.1186/s12913-018-2910-7

**Published:** 2018-02-13

**Authors:** Yangjing Bai, Jiping Li, Yangjuan Bai, Weiguang Ma, Xiangyu Yang, Fang Ma

**Affiliations:** 1 Department of Cardiovascular Surgery, West China Hospital, Sichuan University, Chengdu, China; 2 Department of Nursing, West China Hospital, Sichuan University, Chengdu, China; 3 Cardiology Department, The First Affiliated Hospital of Kunming Medical University, Kunming, China; 4 School of Nursing Peking Union Medical College, Beijing, China; 5 School of Nursing, Chengdu University Of Traditional Chinese Medicine, Chengdu, China; 6 Department of Nursing, The First Affiliated Hospital of Kunming Medical University, 295#, Xichang Road, Kunming, China

**Keywords:** Training transfer, Nurses, Validity, Reliability

## Abstract

**Background:**

Most organizations invest in people for training to improve human capital and maximize profitability. Yet it is reported in industry and nursing as well that training effectiveness is constrained because of inadequate transfer of training and the underlying reasons for the transfer problem remain unknown. And there is lack of tool to measure transfer problem.

**Methods:**

The purpose of this study was to develop and validate a questionnaire to evaluate the scores of factors influencing training transfer (FITT) among nursing professionals. The questionnaire was developed by item generation through interview with nurses and literature review. The FITT was validated in terms of content validity through expert reviews. Psychometric properties of the final instrument were assessed in a sample of 960 nurses with training experiences.

**Results:**

The content validity of the instrument were as follows: the IR was 0.8095. 51 items on the 63-item scale had I-CVIs of 1.0 and the remaining 12 items had I-CVIs of 0.88. The S-CVI/UA was 0.976 and the S-CVI/Ave was 0.977. For the exploratory step, principal axis factoring (PAF) was selected for this study. Parallel analysis was used to decide the number of factors to extract and oblimin rotation method was used. Exploratory factor analysis identified a five-factor solution including 53 items, accounting for 68.23% of the total variance. The confirmatory factor analysis showed some support for this five-factor model. The findings demonstrate high internal consistency (Cronbach’s alpha = .965).

**Conclusions:**

This study indicates that the FITT is a valid and reliable instrument for assessing the factors influencing training transfer among nursing professionals. The FITT can be used to assess individual perceptions of catalysts and barriers to the transfer of training among nursing professionals, which can help promote training transfer and training effectiveness in the workplace.

## Background

With the shelf life of new knowledge growing shorter and job positions changing almost overnight, health care professionals have always been encouraged to update their knowledge and maintain clinical competence to meet new demands [1]. Without a program of active learning no health care professional can hope to remain competent for more than a few years after graduation [2], which increases the pressure of health care professionals to engage in training and education. To survive in the increasingly competitive markets, health care organizations need to strengthen internal staff training and development programs to have employees with highly developed skills [1]. Successful training in health care institutions can lead to professionals’ enhanced knowledge and skills, improved retention and recruitment, reduced patients’ mortality and high-quality patient care [1, 3–5]. It is undoubtedly that training can contribute significantly to organizational and personal success. The need for training in our health care institutions is expanding exponentially, yet the dollars to support such training are not [1]. Exacerbating the problem^.^is the research findings which show that despite the billions of dollars and employee hours that organizations dedicate to education and training program, there is limited evidence that knowledge and skills learned during these programs are transferred to the workplace [6]. One estimate suggests that employees transfer less than 10% of training and development expenditures back to their workplace [7]. To get maximum value from the training dollars, it is crucial to improve the effectiveness of training and get the best use of training funds.

Along with the large training efforts that have been invested and the low pay-off of training expenditure in on-the-job performance improvements resulting from the transfer of learned knowledge, skills, and abilities in today’s global organizations, has come substantial research attention devoted to understanding and improving training effectiveness. According to Kirkpatrick, the key criterion for evaluating training effectiveness is transfer of training [8, 9]. Transfer of training is defined as the extent to which knowledge, skills and attitudes learned in work-related training are applied on the job and subsequent maintenance of them over a certain period of time [10]. To make sure that the training is effective, and to get value from the time and money invested in training, the trainees must apply what they learned in training on the job or positive training transfer occurs [11]. The first step to improve training transfer is an accurate diagnosis of those factors that are inhibiting it. Therefore, developing a comprehensive, generalizable, valid instrument of factors that influence training transfer will help organizations effectively and efficiently manage transfer interventions by diagnosing the strengths and weaknesses of the key factors that affect the transfer of training. Finally, the return on investment from training investments can be enhanced.

Baldwin and Ford [12] proposed a model which indicates transfer of training is traditionally seen as a function of three factors: trainee characteristic, including ability, personality, and motivation; training design, including principle of learning, sequencing and training content; and work environment, including support and opportunity to use. Since then, researchers have generally viewed training transfer as been affected by a system of influences which continue to fall within the three broad categories of the trainee characteristics, training design, and work environment factors [13]. Accordingly, a wide variety of measures have been used to assess various factors affecting training transfer but most of them have questionable psychometric qualities [14]. Rouiller & Goldstein [15] developed a tool to investigate the organizational transfer climate and suggested that it was a potential facilitator for enhancing positive transfer of training into the work environment. However, they were unable to validate the construct structure of the scale and it only measures some environmental factors [14]. Tracy & Tews [16] developed a general training climate scale (GTCS) to examine the factors influencing training transfer and examined its construct validity, yet the scale is mainly concerned about the environmental factors. Holton et al. [14, 17] developed a Learning Transfer System Inventory (LTSI) to measure a select set of factors with the potential to substantially enhance or inhibit transfer of training to the work environment. It has undergone a variety of validation studies and has been applied in many situations, but there were some discrepancies in factor solutions in some studies together with problematic fit of particular items, such as a disproportionate number of items across factors and low internal consistency reliability in some factors. Besides, factor stability in different types of organizations and training interventions is not fully examined and to enhance its practicality the LTSI should be made more parsimonious [9, 10, 18–20]. Furthermore, in the development and validation of this inventory, most studies were conducted in industry and business, whether it is applicable in nursing profession remains questionable. To date, no tool has emerged to diagnose the factors that affect training transfer among nursing professionals. The lack of a well-validated and reasonably comprehensive set of scales to measure factors influencing training transfer among nursing professionals may be a key barrier to improving training transfer and training effectiveness among nursing professionals. The purpose of this study is to develop and validate a questionnaire to evaluate the factors influencing training transfer (FITT) among nursing professionals.

## Methods

### Study design

A mixed-method design combing a qualitative study with a quantitative procedure was utilized. The instrument to evaluate the factors influencing training transfer (FITT) among nursing professionals was developed in two phases: Phase One for item generation and development of the questionnaire, through interviewing nurses, expert judgments and content validity testing by manager experts; and Phase Two for testing other psychometric properties, such as construct validity and internal consistency reliability. The study was approved by the Kunming Medical University Research Ethics Committee. Participants in qualitative and quantitative study were given written information about the aim and the procedures of the study, and the right to withdraw at any time. Participants were assured that their names would not be used, and confidentiality would be maintained by the researchers. Before data collection informed consent was obtained from each participant. Participation was voluntary.

### Phase I -Qualitative Phase/Development of the FITT instrument

This phase included two stages: (1) Development of an item pool; (2) Item reduction and development of the FITT.

#### Development of an item pool

##### Procedure and participants

A qualitative study using in-depth, face-to-face interviews was adopted in this study, which aimed to explore nurses’ perspectives on the factors influencing training transfer among nursing professionals. The purposive sampling was adopted and participants were nursing staff and managers at two large hospitals in south west China. Twenty-one nurses and three nurse managers in charge of training completed a semi-structured interview between February 2013 and September 2013. The following topics guided the interviews: (1) Recall your training experiences home and abroad, did you apply the new knowledge and skills learned in training on your work environment and what supported or hindered you? (2) Considering your training experiences as trainers, trainees, and training managers, what factors facilitate and hamper the application of what learned in the training on the workplace. The data were analyzed using qualitative content analysis [21, 22]. The model of the transfer process presented by Baldwin and Ford was used to guide the analysis [12], which could inform the initial direction of qualitative data analysis without limiting the identification of new themes.

The above qualitative findings provided directions for developing a practical assessment tool to identify the factors influencing training transfer among nursing professionals. The themes that emerged from the qualitative data were used to build on the related constructs, which have provided the basis for the development of scale items. With the interview data and a critical review of existing literature and related assessment tools on factors influencing training transfer, the first draft of the item pool was developed. According to DeVellis’ [23] recommendations, effort was made to write all statements in a way that was clear, understandable and unambiguous to the respondents. In order to minimize the possibility of set responses, items were written in both positive and negative directions.

Ten themes emerged from the qualitative analysis data: motivation; ability; self-efficacy; value; training content; instruction method; support; opposition; transfer climate; professional development. We developed an item pool with 190 items according to the qualitative results and literature review.

#### Item reduction and development of the FITT

##### Procedure and participants

The 190 items pool was validated by a four-member expert panel selected for their expertise in the areas of nursing management [24]. Criteria used by the judges for retention or deletion of items included clarity of expression, face validity, appropriateness for the construct being measured, and potential for differentiating the target population [23]. Each expert indicated her decision (to remove, keep, or modify) for each item and made comments for the modified items. Items which were consistently judged to be removed were eliminated and modification was made to the modified items. After item reduction and modification, the left items were used for the first draft of FITT, which was again sent to eight experts for validation of face and content validity. These experts included two directors of nursing department with PhD qualifications and specializing in nursing administration; one vice president of the hospital and three vice directors of nursing department with rich experiences in nursing administration; two business-administration professionals with PhD qualifications and experiences of human resource management. The experts offered comments and suggestions about whether some items should be added, removed or modified. They also evaluated the level of relevance of each item for its corresponding construct on a 4-point scale (1 = not relevant, 2 = somewhat relevant, 3 = quite relevant, 4 = highly relevant) [25, 26]. The interrater agreement(IR); content validity index for items(I-CVI); scale-level content validity index, universal agreement calculation (S-CVI/UA); scale-level content validity index, averaging calculation method (S-CVI/Ave) were computed to indicate the content validity [26–28].

Based on the four-member experts’ suggestions, 63 items were retained after the item reduction and some items were modified to be more readable and explicit. These items were used for the first draft of the FITT. In the validation of face and content validity process, according to eight experts’ comments and responses, there were no additional new items, no deletion of any items or further modifications to the FITT. The IR was 0.8095. 51 items on the 63-item scale had I-CVIs of 1.0 and the remaining 12 items had I-CVIs of 0.88. The S-CVI/UA was 0.976 and the S-CVI/Ave was 0.977, which meant the items were good operationalizations of the underlying construct.

A two-part questionnaire was developed on the basis of the 63 items. The first part asked respondents to describe their personal characteristics, including age, gender, department etc. The second part consisted of 63 items to which responses were given using a 7-point Likert scale (1 = strongly disagree, 7 = strongly agree). This part were subdivided into two sections: Training in Specific and Training in General. Section A (Training in Specific) contained 48 items that measuring 7 constructs and focused on “the specific training program”; section B(Training in General) contained 15 items that measuring 3 constructs and focused on “training in general”.

### Phase II -Quantitative Phase/Psychometrics properties of the FITT questionnaire

This phase included two stages: (1) Pilot study; (2) Validation of the FITT questionnaire.

#### Pilot study

The first version of the FITT questionnaire (63 items) was given to a convenience sample of 50 nurses who had attended a PICC(Peripherally Inserted Central Catheter) training program a month ago to evaluate item clarity and to estimate reliability. Nurses’ comments revealed no lack of clarity in the wording of the items. The items were readable, explicit and accurate in reflecting the factors influencing training transfer among nursing professionals. Data were analyzed for internal consistency and the Cronbach’s coefficient alpha was 0.972, indicating high internal consistence [29]. The instrument appeared to have sufficient reliability and warrant further development.

#### Validation of the FITT questionnaire

##### Instrument

The first version of the FITT is a 63-item self-report survey designed to evaluate individual perceptions of barriers and catalysts to training transfer among nursing professionals. Each item is rated on a 7-point Likert scale ranging from one (strongly disagree) to seven (strongly agree).

#### Sample

Eligible subjects were nurses with experiences of job related training. In the selection of participants, two aspects should be considered as for the time period between the end of training and the survey. On the one hand data should be collected at a certain period of time after training to enable the trainees to respond to the items in the instrument according to their actual experiences, such as items measuring supervisor and peer support; on the other hand to avoid trainees’ forgetfulness of the training experience and subsequent inaccurate response to the items, the time period should not be too long. According to related researches [9, 30–34], we considered the appropriate time period between the end of training and the survey should be one to 3 months. Nurses who attended a training program one to 3 months ago were included in our survey. In the process of completing Section A of the questionnaire, participants were asked to respond to the items in terms of their perception of the training program they had attended recently, whereas in completing Section B of the questionnaire, they were asked to respond to the items based on their general impression of training in their organization.

The number of nurses used to validate this tool was calculated based on an item to participant ratio of 1: 5 to 1:10 [35, 36]. We included more nurses in consideration of missing data. Because the purpose of this survey was to develop a generalized instrument that could be used across a wide range of training programs and organizations among nursing professionals, the sample was deliberately chosen to be as heterogeneous as possible. The sampling strategy was purposive and the FITT was administered to 960 nurses who attended a wide variety of training programs one to 3 months ago.

#### Data collection

The survey was conducted between February 2014 and April 2014 in ten different hospitals in south west China. Data were collected by the researchers, using questionnaires, at nurse meetings in hospital units and by email. Questionnaire completion was voluntary and anonymous. The response rate across the hospitals was 95.42%; a total of 916 questionnaires were returned and analyzed.

#### Data analysis

The validity and reliability of the FITT were evaluated as follows: Construct validity was established by exploratory factor analysis (EFA) and confirmatory factor analysis (CFA). The entire study sample (*n* = 916) was divided into two sub-samples randomly (A and B). The factor structure of the FITT was first examined by EFA in sub-sample A (*n* = 458). Values for Kaiser–Meyer–Olkin (KMO) measure of sampling adequacy and Bartlett’s test of sphericity (preferably significant) were used to assess the suitability of data for factorisation. EFA was used to explore the common factors in the latent variable using SPSS 20.0. Principal axis factoring (PAF) was selected for this study, which aimed to explore the theory of training transfer system rather than data reduction. Parallel analysis was used to decide the number of factors to extract and oblimin rotation method was used to allow meaningful components to be identified. The criterion for loading and cross loading was set at 0.4, and based on this, items with loading below 0.4 and cross loading over 0.4 were deleted. This process was repeated until a simple structure was achieved where loadings were maximized on putative factors and minimized on the others [37–39]. At least three variables per factor were required to identify factors that were stable [40]. Confirmatory Factor Analysis (CFA) was used to cross-validate the factor structure derived from EFA of the FITT. To further corroborate the stability of the factor structure, the data from sub-sample B (*n* = 458) were used for CFA.

A number of indices of model fit were used to assess the goodness of fit of the CFA model. The indices utilized in this study were root mean square error of approximation (RMSEA), comparative fit index (CFI), standardized root mean square residual (SRMR), incremental fit index (IFI) etc. [41]. The internal consistency was established by calculating Cronbach’s alpha coefficients. Statistical analysis was undertaken using the Statistical Package for the Social Sciences, version 20.0 and Advanced MOrtar System (AMOS), version 21.

## Results

### Demographic data

Of the 916 nurses who submitted completed questionnaires, 35 (3.82%) were male and 881 (96.18%) were female. A total of 602 (65.72%) were of Han nationality and 314 (34.28%) belonged to other ethnic groups. As for the Level of education, 493 (53.82%) had baccalaureate degree or above and 423 (46.18%) had associate degree or below. The training program covered a wide variety of topics (Table 1).

**Table 1:** Sample information by training (*n* = 916)

Training	Types of hospital	Frequency	Percentage	Cumulative Percentage
Closed sputum suction	Large/provincial	43	4.7	4.7
Care and Maintenance of CVC (central venous catheter)	Large/provincial	22	2.4	7.1
Modes and parameters of mechanical ventilator	Large/municipal	25	2.7	9.8
First-aid technique	Small/municipal	99	10.8	20.6
Nursing management	Large/provincial	117	12.8	33.4
Patient safety	Large/provincial	47	5.1	38.5
Policy and procedure in nursing work	Large/provincial	75	8.2	46.7
Nursing documentation	Small/municipal	26	2.8	49.5
Laws and regulations in health care setting	Small/municipal	24	2.6	52.1
Health education knowledge	Medium/municipal	80	8.7	60.8
Infection control and occupational protection	Large/municipal	50	5.5	66.3
Specialist nurse knowledge	Large/municipal	103	11.2	77.5
Professional ethics and etiquette	Large/municipal	36	4.0	81.5
Communication skills	Small/county	49	5.4	86.9
Nursing education	Large/provincial	25	2.7	89.6
Nursing research	Large/municipal	20	2.2	91.8
Clinical nurse specialist training (emergency)	Medium/municipal	35	3.8	95.6
Clinical nurse specialist training (wound and ostomy)	Large/provincial	40	4.4	100

Types of hospital: large hospital: more than 1000 beds; medium hospital: 500 < beds<=1000; small hospital: beds<=500; provincial hospital: supervised by provincial government; municipal hospital: supervised by municipal government; county hospital: supervised by county government

### Exploratory factor analysis

The 63 items were subjected to EFA to examine the factorial validity of the scale. The Kaiser-Meyer-Olkin measure of sampling adequacy was.970 and Bartlett’s test of sphericity reached statistical significance (*P* = .000), which suggested that these data very suitable for factor analysis [42, 43] . A five-factor solution including 53 items and explaining 68.23% of the total item variance in the database was obtained. Factor 1 (20 items), “managerial support” accounted for 46.973% of the variance; Factor 2 (6 items), “hindrances in the organization” accounted for 7.149%; Factor 3 (10 items), “validity of training program” accounted for 5.968%; Factor 4(11 items) “organizational and personal facilitators”, accounted for 4.586%; Factor 5 (6 items) “personal attitude toward training transfer” accounted for 3.554%. Table 2 presents the items with their loadings in each factor.

**Table 2:** Rotated factor loadings of the FITT questionnaire items

Item	Factor Loadings	Variance explained
Factor 1: managerial support		46.973%
36. After the training, my supervisor gives me opportunity to use what I learned in the training.	.880	
38. My supervisor meets with me to work on problems I have in trying to use my training.	.879	
37.After the training, my supervisor gives me resources for applying what I learned in the training.	.872	
40. My supervisor rewards or punishes me based on my use of what I learned in the training.	.846	
35. My supervisor encourages me to use what I learned in the training effectively.	.800	
34. After the training, my supervisor sets a realistic goal for job performance based on my training.	.748	
39. My supervisor supervises my use of the training in the process of applying what I learned in the training.	.735	
33. After the training, my supervisor meets with me to discuss ways to apply the training on the job effectively.	.712	
41. My organization set goals for me to apply my training on the job before the training.	.694	
46. My organization gives me resources for applying what I learned in the training.	.688	
45. After the training, my organization gives me opportunity to use what I learned in the training.	.684	
48. My organization provides opportunity for me to update my training in the process of applying what I learned in the training.	.664	
23. If I successfully use the training in the workplace, I will get affirmation and reward.	.655	
47. The related departments give me support and coordinate with me in the process of applying what I learned in the training.	.627	
28. When I try to use the training in the workplace, my colleagues trust me.	.510	
22. If I successfully use the training in the workplace, I will get higher performance.	.483	
43. My organization rewards or punishes me based on my use of what I learned in the training.	.470	
26.My colleagues show interest in what I learned in the training.	.464	
27.My colleagues encourage me to use the knowledge and skills I have learned in the training.	.463	
29. The collaboration among my colleagues is satisfactory when I apply the training.	.403	
Factor 2: hindrances in the organization		7.149%
31. If I try to use the training, my colleagues give me cynicism.	.755	
61. In my organization, my profession is not recognized.	.744	
62. In my organization, my professional development is limited, which makes me hard to use what I learned in training.	.737	
32. When I try to use the training, my colleagues persuade my supervisor not to support my use of the training.	.716	
30. My colleagues have a strong aversion to the use of what I learned in the training.	.707	
63. It is hard for me to combine my career planning with training due to the limited professional development.	.627	
Factor 3: validity of training program		5.968%
6. The interactive atmosphere in the training could help me grasp the training content.	.872	
5. The training method was versatile and flexible, which helped me improve my learning efficiency.	.860	
7. The training method was practice-oriented, which helped me apply my learning on the job easily.	.848	
8. The training was trainee-centered, which facilitated my grasp of the training content.	.790	
4. The training will help me resolve the substantive matters in the workplace.	.752	
9. The trainer gave me evaluation and feedback about my learning after the training.	.731	
3. The training focused on the problems to be resolved in the workplace.	.716	
2. The training helps me improve my work capability.	.660	
1. The training matches my work requirements.	.640	
10. Prior to the training, I was clear about the purpose and request of the training.	.488	
Factor 4: organizational and personal facilitators		4.586%
53. There is an active and enterprising spirit in my organization and the pursuit of knowledge is highly valued.	.870	
52. People in my organization are positive and hope to improve themselves.	.865	
55. In my organization my colleagues are willing to share their knowledge and experiences, collaborate and grow in the work together.	.836	
54. In my organization, colleagues are willing to share what they have learned in the training.	.831	
59. There is an atmosphere of support and acceptance in my organization.	.814	
58. When people encounter frustrations in the process of applying what they learned in training, my colleagues positively support them instead of counteracting their efforts.	.760	
60. People in my organization show tolerance for colleagues who made mistakes in the process of applying what they learned in training.	.707	
51. I feel confident that I can use what I learned in training effectively and resolve the problem.	.706	
56. My organization is open to change and advocates creativity.	.692	
49. I am confident in my ability to use what I learned in training if I try hard enough.	.657	
50. I am sure I can overcome obstacles on the job that hinder my use of what I learned in training.	.651	
Factor 5: personal attitude toward training transfer		3.554%
15. I believe the training should be shared and applied in the organization.	.615	
14. The purpose of my attendance of the training is to resolve the problem in the workplace with the use of my training.	.562	
17. I have a duty to use the training in the workplace effectively after the training.	.555	
16. The application of the training is good for my personal development.	.549	
12. I learned actively in the training because I treasure this training opportunity.	.452	
18. Effective use of the training meets the requirement of the development of my organization.	.413	

### Confirmatory factor analysis

A confirmatory factor analysis (CFA) with maximum likelihood method was used for the other survey cases (*n* = 458) and resulted in the same five-factor structure. Several CFA fit indices (CMIN/DF = 7.553, RMSEA = 0.079, SRMR = 0.053, CFI = 0.902, GFI = 0.702, TLI = 0.784, IFI = 0.902, RFI = 0.859, NFI = 0.879, NNFI = 0.879) indicated moderately good fit for the model [42, 44, 45]. These indices provided confirmatory evidence for the factor structure.

### Internal consistency reliability

The results of Cronbach’s Alpha tests showed that the alpha coefficient of the FITT was 0.965 and five dimensions of Cronbach’s alpha was 0.964, 0.869, 0.958, 0.953, 0.940 respectively, which indicated acceptable internal reliability in both the instrument and the sub-dimensions [29].

## Discussion

To the knowledge of the authors, this is the first study both to develop and undertake a detailed validation of a questionnaire to assess the factors influencing training transfer among nursing professionals. It consists of 53 items, which are organized into five subscales (managerial support, hindrances in the organization, validity of training program, organizational and personal facilitators, personal attitude toward training transfer). In the development of the item pool, the themes emerged from the qualitative findings were used to build on the related constructs and guided the development of the item pool. The draft of the item pool was based on a critical review of existing related literature and assessment tools as well as the interview data in the qualitative phase. The items were generated from the perspective of nurses and verified by the published literature that specialized in the field, this approach ensured the content validity of the tool at the beginning of the research. The process would also help fine-tune the language and relevance of the developing instrument, which is becoming more popular and highly recommended by researchers [46]. In the development of the questionnaire, the developed item pool was validated by expert panel selected for their expertise in the areas of training management, which could strengthen the face and content validity of the questionnaire.

In the assessment of the psychometrics properties of the FITT questionnaire, the evidence for the construct validity of the FITT was supported by exploratory and confirmatory factor analysis. In the study, EFA of the FITT yielded a five-factor model that explained 68.23% of the variance in the study. CFA was used to validate the EFA derived factor structures of the FITT. Results indicated moderately good fit for the instrument, offering confirmatory evidence for the factor structure. An RMSEA of less than 0.06 is considered a close fit, while values between 0.06 and 0.08 are considered an acceptable fit [45]. The values of NFI, NNFI, IFI, RFI, GFI, CFI and SRMR supported the acceptable fit of the model. In this study, the internal consistency of the FITT and its 5 subscales, as measured by Cronbach’s alpha, were all greater than 0.869. They were highly satisfactory [47]. Through the purposive sampling technique, the sample data used in these analyses came from a wide range of organizations and training programs, we believe that the sample collected from this study reached a level of heterogeneity that its generalizability is vastly improved.

Given these findings, the FITT can be considered as a valid and reliable instrument in assessing the factors influencing training transfer among nursing professionals. As training research and practice move beyond the question of whether or not training works to why training works and how the transfer outcomes and training effectiveness can be improved, measurement of factors affecting training transfer among nursing professionals will become more important. In practice, the FITT can be utilized as a “pulse-taking” diagnostic tool for investigating known transfer of training problems. According to the assessment of the FITT, interventions can be designed to enhance training transfer among nursing professionals.

### Limitations

This study represented an initial step in the development and validation of the FITT. Several limitations of the instrument must be considered, such as a disproportionate number of items across factors. Effort should be made to reduce the size of the instrument to keep it parsimonious while retaining the factor structure and its psychometric quality. Another limitation of the study is the inclusion of nurses from south west China, and hospitals in different locations have different cultures and organizational atmospheres, which may lead to diversity among nurses and the sample can’t be representative of all nurses. Further studies may use the FITT among nurses in a wide variety of institutions and training programs.

## Conclusions

The FITT is a reliable and valid instrument for assessing the factors influencing training transfer among nursing professionals. Although more research is needed to strengthen the future development of the FITT, preliminary findings suggest that this tool is a well-validated and reasonably comprehensive instrument for diagnosing the factors that affect training transfer among nursing professionals, which measures related factors influencing training transfer that include personal, training and environment aspects. In this research, factor stability in different types of organizations and training interventions among nursing professionals were examined and which shows a high internal consistency reliability in all the factors. The assessment process and outcome can enable nurse administrator to identify the enablers and hindrance of training transfer among nursing professionals, which provides valuable information for the improvement of training effectiveness.

## Abbreviations

CFA: Confirmatory Factor Analysis
EFA: Exploratory Factor Analysis
FITT: Factors Influencing Training Transfer
PAF: Principal Axis Factoring
